# Micro-Crack Mechanism in the Fracture Evolution of Saturated Granite and Enlightenment to the Precursors of Instability

**DOI:** 10.3390/s20164595

**Published:** 2020-08-16

**Authors:** Longjun Dong, Yihan Zhang, Ju Ma

**Affiliations:** School of Resources and Safety Engineering, Central South University, Changsha 410083, China; lj.dong@csu.edu.cn (L.D.); zyh6324@csu.edu.cn (Y.Z.)

**Keywords:** acoustic emission, sensor, RA-AF, moment tensor, source type, micro-failure precursor

## Abstract

To explore the potential precursors of rock instability, it is necessary to clarify the mechanism of micro-crack from fracturing to failure, which involves the evolution of fracture size, orientation, source model, and their relationships to the loading. The waveforms of acoustic emission (AE) recorded by the sensor network attached rock sample during laboratory tests provide a data basis for solving these problems, since these observations are directly related to the characteristics of the fracturing sources. Firstly, we investigated the source mechanism, looking at the rise angle and the average frequency (RA-AF) trends during five loading stages in a uniaxial compression test. Results show that the proportion of shear events significantly increases when approaching instability. Secondly, we calculated the moment tensor for each event, considering the uncertainties of P-wave polarity, azimuth, and the takeoff angles of the rays. Moment tensor solutions suggest that there are obviously more crack events than shear events in all loading stages. Moment tensor evolutions confirmed that the decreasing of isotropic component and the increment of double-couple can be used as precursors of rock fracturing development. Considering the limitations of these two methods, it is suggested that we should be concerned more about the proportions of individual failure components and their evolutions over time, instead of absolutely classifying the events into a certain source type.

## 1. Introduction

The macroscopic failure of the rock is a result of a series of complex and diverse micro-cracks accumulation, nucleation, and interconnection. The models of rock micro-cracking under compressive stress include grain crushing, sliding along pre-existing cracks, stress concentrations around pores, elastic mismatch between grains, dislocation movement, and combinations of these mechanisms [1]. These micro-cracking models not only respectively dominate the rock fracturing process at different stages of loading, but also demonstrate the fracturing scale and type. From the perspective of fracture mechanics, rock cracks are usually characterized as a combination of basic types of tension and shear [2]. Exploring the rock fracture mechanism is helpful to understand the causal mechanism of rock engineering disasters, which plays an important role in predicting, controlling, and reducing emergencies.

Acoustic emission (AE) analysis provide spatial-temporal information about the evolution process of rock fracture [3,4,5,6], where the AE waveforms emitted under different fracture modes have obvious differences [7]. Combining the time domain and frequency domain characteristics of AE signals is a significant means to characterize rock fracture types. Generally, tensile crack forms AE waveforms with shorter rise time and higher average frequency, whereas, the shearing process always lead to the AE waveforms with longer duration, lower average frequency, and more delayed maximum amplitude [8,9].

In the time domain, the gradients of an ascending part of the AE waveform can evaluate the evolution process of different rock cracks in shear and bending test, which relate to three types of rock fracture: tension, shear, and mixing [7]. The gradient of the waveform rise refers to the ratio of the rise time to the amplitude (RA). In frequency domain, the change of AE frequency not only reveals the size of the crack scale [10], but also the fracture type of AE source. The statistical analysis of the AE dominant frequency is meaningful for understanding the microscopic failure mechanism of rocks. Surrounding the phenomenon of dual dominant frequency bands in the process of rock fracture, the AE signals of high frequency and low frequency are considered to be generated by shear and tension fracture, respectively [11]. Based on the above-mentioned basic viewpoints, some researchers have discussed the evolution process of shear and tension cracks in white marble under direct tensile test [11], uniaxial compression [12], direct tensile test and Brazilian test [13]. The dominant frequency identification method is referential, but the complexity of the rock fracture mechanism should still be considered. The main energy of a micro-fracture is not necessarily all concentrated in the dominant frequency [14], and the entire fracture process exists in more than two dominant frequency bands [15]. Combining the perspectives of time domain and frequency domain, the classification of concrete crack types by the rise angle and the average frequency (RA-AF) characteristic parameters has been extensively studied and discussed, and the analysis method of RA-AF characteristic parameters has been verified by SiGMA analysis [16,17]. This method has gradually developed to the definition of crack types in different rocks under various mechanical environments [18,19,20]. However, there is still a lack of quantitative characterization methods to determine the transition line between the two types of fracture. The existing empirical methods include: (1) the test division method to carry out a single fracture type-led test, such as three-point bending tests, direct shear tests, and determine the transition line of the fracture type is determined by the RA-AF values distribution of the AE signal in each test [19,21]; (2) the dominant frequency feature division method is based on the AE dominant frequency to define the type of shear and tension fracture [22]. 

In addition to the methods mentioned above, there are many studies using moment tensor to identify the type of rock failure [23,24,25,26,27]. Moment tensor is a matrix of nine force couples describing the state of stress at the source. Cautious analysis of the moment tensor matrix makes it possible to infer the rock mass fracture process. Zhang et al. [23] classified the microfractures into five types (compression, shear-compression, shear, shear-tensile, tensile), based on the tensile angle derived from moment tensor interpretation. Yamamoto et al. [24] classified hydraulic fracturing into shear-dominant events and tensile/compression-dominant events, by the consistency coefficient, which is a function of the classic moment tensor decomposition (isotropic (ISO), double-couple (DC), and compensated linear vector dipole (CLVD)). Hampton et al. [28] categorized AE into shear, tensile, and mixed mode, through manipulating the eigenvalues of the moment tensor matrix. This method has been widely used to identify rock failure under different stress conditions [29,30,31]. Actually, this method was first proposed for an in situ hydrofracturing test [32]. The author developed this method [33] and compared it to the parameter-based method [16].

The classification threshold, based on waveform-frequency features, is usually derived from empirical statistics, and the moment tensor analysis method also brings uncertainties, due to inaccurate P-arrival picking. To quantify the uncertainties of different methods and determine the evolution of microfracture mechanism from nucleation to failure, we evaluated the evolution of different types of cracks in granite under uniaxial compression, using moment tensor inversion and AE characteristic parameters.

## 2. Experiment

### 2.1. Instruments and Rock Samples

**Figure 1:**
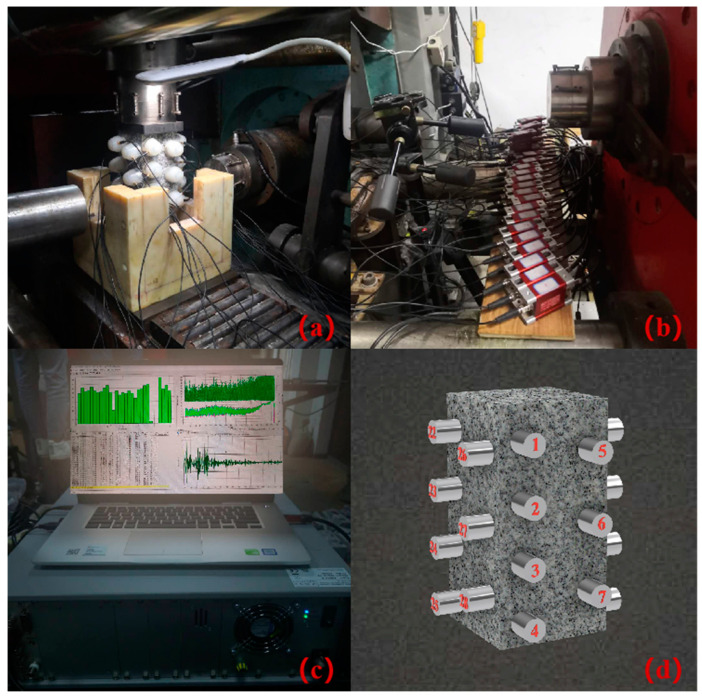
Testing system and Sensor arrangement: (**a**); Sample, AE sensor, and loading device; (**b**) Preamplifier; (**c**) AE collection interface and host machine; (**d**) AE sensor layout.

The experimental instruments include two parts: stress loading equipment and AE monitoring system (Figure 1). The loading equipment is the true triaxial electro-hydraulic servo mutagenesis test system with model TRW-3000, which can perform dynamic and static rock mechanics experiments on samples of various specifications. The test system can achieve independent loading in both vertical and horizontal directions and the uniaxial compression test is carried out using the vertical loading frame during the experiment. It records load, stress, displacement and strain values in real time, and simultaneously draws load-displacement, stress-strain curves. An AE monitoring system equipped with the highest standard international acquisition card is AMSY-6 multi-channel equipment from Vallen, Germany (consisting of parallel measuring channels) and achieves synchronous AE characteristic parameters and waveform acquisition during the experiment. Each block has 2 independent channels. The channel ADC, the accuracy, and the wideband operating frequency are 40 MHz, 18 bits, 18 KHz–2.4 MHz respectively. The sensor in the experiment was the VS45-H sensor, with a response frequency of 20 KHz–450 KHz.

Granite, taken from the depth of 100 m underground in the Linglong gold mine, was selected, which is made into a standard cuboid rock sample of 100 mm × 100 mm × 200 mm. The surface of the rock sample meets the parallelism, flatness, and perpendicularity requirements of ISRM recommendations. Water is added by 1/4 gradient in the process of soaking granite to make saturated samples. In order to minimize the differences among samples, all samples are taken from one granite rock.

### 2.2. Sensor Arrangement and Loading Procedure

In the experiment, 28 AE sensors are arranged on the surface of the rock sample, utilizing alternating dislocation. The sensor is fixed by a fixing device, to avoid falling off during the loading process, and using a couplant in the contact area between the sample and the sensor for the sake of the coupling effect. According to the site environment of the experiment, the pre-amplifier, pre-filter, threshold, and sampling frequency of the AE experimental analysis system are set to 40 dB, 95–450 KHz, 55 dB, 10 MHz, respectively.

Before the experiment, the hidden problems in the experiment were eliminated by checking the installation of the sample, the coupling degree of AE sensor and debugging the equipment. The stress loading rate set in the uniaxial compression experiment is 500 N/s. A preload of 1–2 KN is applied to the rock sample to ensure that the rock sample is in full contact with the upper compression plate, to eliminate the noise generated when the rock sample contacts the plate. The loading test system and AE monitoring system are synchronized, and the actual data are recorded in real time during the experiment. The experiment was carried out until the granite lost bearing capacity completely.

## 3. Results

### 3.1. AE Time-Frequency Characteristics of Granite Damage Evolution

A single AE event triggers multiple channels to record AE hits at the same time, the AE waveforms and parameter characteristics of which are often highly consistent. We define the characteristic parameter value of AE event as the average value of its corresponding characteristic parameter value of AE hits. AE event rate and amplitude respectively represent the degree and scale of micro-fracture in the process of granite damage accumulation. The dominant frequency of AE events reflects the type and mechanism of granite damage and fracture. The above characteristic parameters can effectively characterize the progressive state of crack under uniaxial compression.

**Figure 2:**
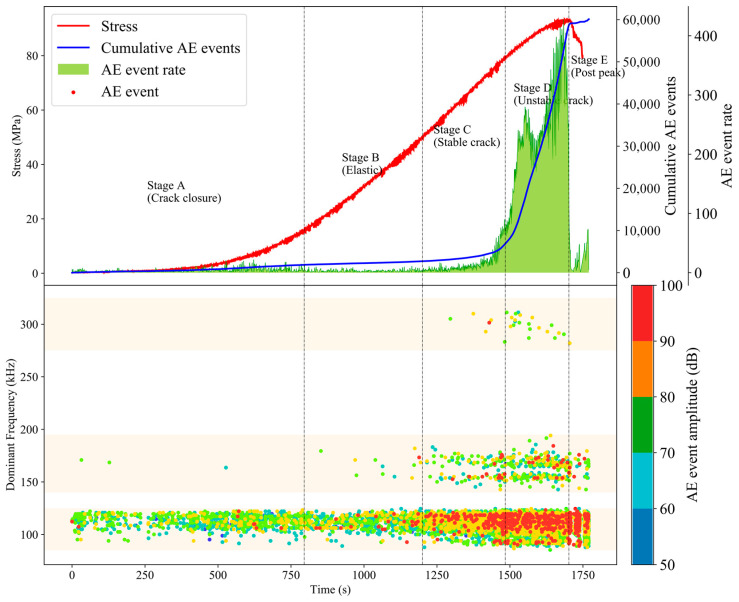
The AE timing and spectrum characteristics of granite in uniaxial compression.

In Figure 2, the accumulation trend of AE events indicates that the initiation, nucleation and expansion of granite fracture are positively correlated with the change of uniaxial stress loading, and the AE amplitude is distributed to the high amplitude interval during the process of uniaxial compression of granite damage evolution. The dominant frequency of AE events appears in three continuous dense bands: high frequency band (280 KHz–320 KHz), low frequency band (85 KHz–125 KHz), and intermediate frequency band (145 KHz–195 KHz).

According to the evolution laws of stress and AE, the entire process of uniaxial compression of granite can be divided into 5 stages:

Stage A (the compaction stage during 0–795s): the characteristics of AE event are low amplitude, low frequency, and low event rate. The main sources of AE signals originate from the primary cracks closed and the friction dislocation between grains.
Stage B (the elastic stage during 796–1200s): the AE activity is almost quiet, and the characteristics are similar to the previous stage.
Stage C (the stable crack propagation stage during 1201–1484s): the increase of AE event rate is accompanied by a large number of AE events with high amplitude and intermediate and high frequency. The primary cracks and secondary cracks with different scales and multiple fracture modes propagate stably in granite.
Stage D (the unstable crack propagation stage during 1485–1702s): the AE events of high energy level increase sharply, and the low frequency band becomes wider, and the low, intermediate and high frequency bands coexist, which reveal that the crack propagation of granite is accelerated, and microcrack initiation and large crack penetration are synchronous.
Stage E (the post-peak stage during 1703–1770s): AE events with high-frequency basically disappear, and AE events with low-frequency and high-amplitude dominate the rock damage. The crack penetration leads to the formation of discontinuity surfaces inside the granite, and the weakened coupling between the sensor and the rock surface, which causes the attenuation of the acquisition wave frequency, especially the influence on the AE signal with high-frequency. With the further penetration of macroscopic cracks, the energy stored in pre-peak continues to be released, and the granite gradually loses its bearing capacity.

### 3.2. Fracture Types of Granite Damage Evolution

**Figure 3:**
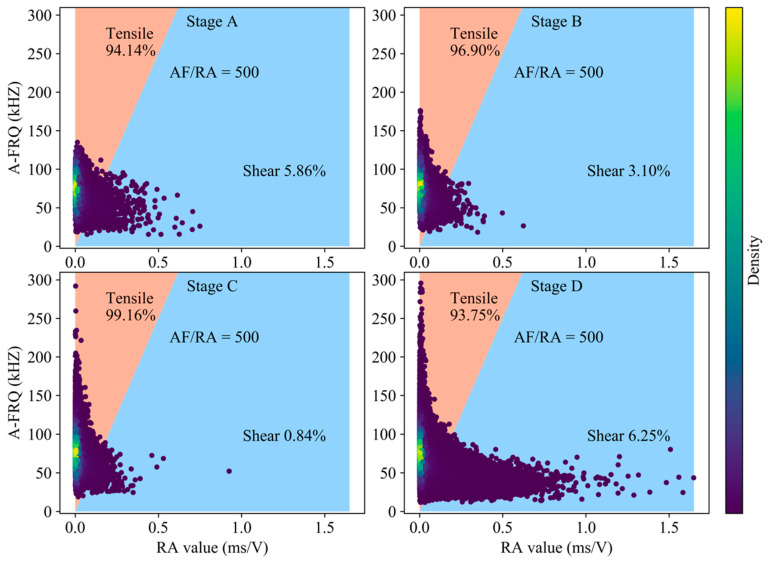
Classification of crack mode based on RA-AF value in the pre-peak stage (using all AE hits).

**Figure 4:**
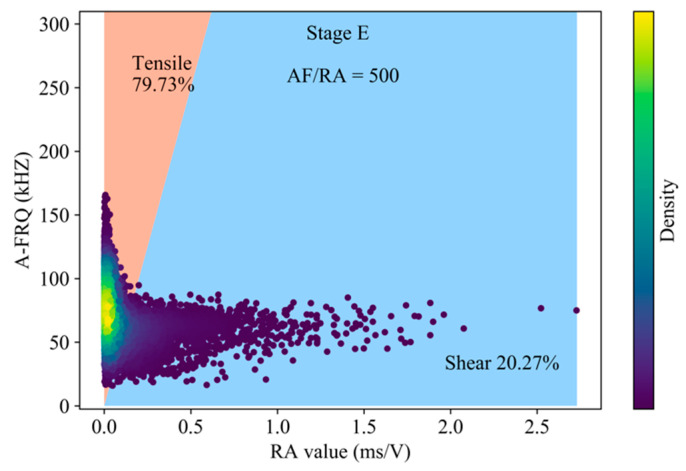
Classification of crack mode based on RA-AF value in the post-peak stage (using all AE hits).

According to the research of Zhang and Deng [12] on the classification of rock crack types, we choose the ratio of RA-AF of 1:500 as the transition line between tension and shear cracks in Figure 3. Tensile cracks are dominant during the generation of cracks in the compaction and elastic stage, and the slip of closed cracks surface and dislocation of crystal are the sources of shear cracks. The proportion of tensile cracks in stable crack propagation stage reaches 98.16%, while the proportion of shear cracks in unstable crack propagation stage and post-peak rises significantly. The tensile strength of rock material is lower than the shear strength, especially in the low lateral restraint state. Entering the stable crack propagation stage, the expansion of granite secondary cracks is dominated by tension, and the occurrence of tension cracks precedes shear cracks. The damage intensifies in the unstable propagation stage, leading to the coalescence of micro fracture and forming a large number of surfaces of discontinuity. Under the action of high axial stress, considering the heterogeneity of rock mass, the deflection of crack development direction and the friction slip between fracture interfaces will generate a large number of shear cracks. The stored strain energy of granite continues to be released after the peak. The propagation of granite cracks is mainly in the form of local fragment rock mass swelling slip and macroscopic fracture surface connecting, and the phenomenon of shear slip has increased sharply compared to the previous 4 stages (Figure 4).

### 3.3. The Influence of Water on the Fracture Types

The influence of water on rocks is mainly reflected in water–rock interaction and pore pressure [34,35]. The natural granite does not appear post-peak stage in that the axial stress drops rapidly after reaching the peak. Therefore, we compared the proportions of crack types of natural and water granite in the four stages under pre-peak (Table 1). Compared with granite in its natural state, the proportion of shear cracks will decrease when saturated granite is about to fracture. This is consistent with Vavryčuk’s research that the moment tensor inversion results show that the water-filled rock has fewer shear components [36]. Water could promote the growth of rock cracks and inhibit the generation of shear cracks.

## 4. Discussion

### 4.1. Uncertainty of the RA-AF Parameter Characteristics Method

**Figure 5:**
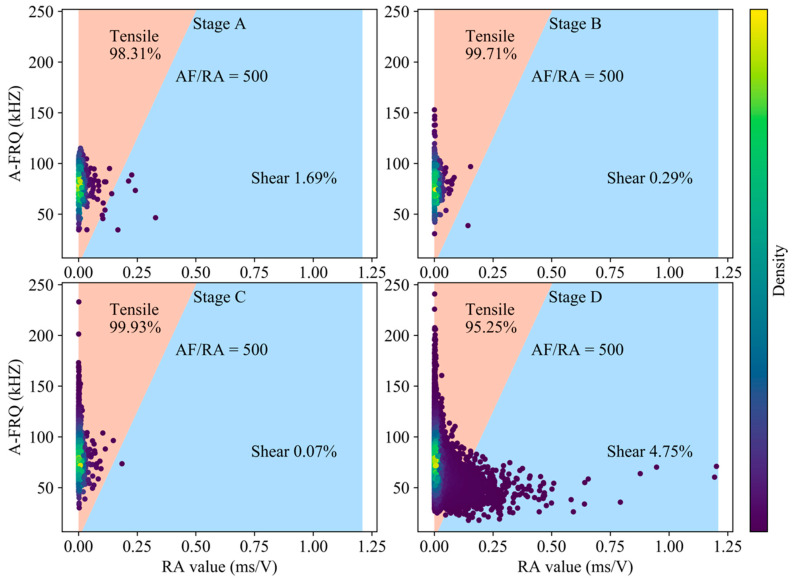
Classification of crack mode based on RA-AF value in the post-peak stage (using first-arrival AE hit).

The influence of AE signals selection should be taken into consideration in fracture type analysis (Ohno and Ohtsu [16]). In Figure 5, the results show that the proportion of shear cracks in each stage decreased, and the increase in the proportion of shear cracks under the entire process is significant when using first-arrival AE hit (FAH). It is recommended to use the first-arrival AE hit to analyze the fracture types when employ the RA-AF parameter characteristics method, whereas, the analysis using all AE hits (AH) causes deviations to a certain extent, since the significant attenuation of the frequency and amplitude of the stress wave leads to the increasement of the RA value and the descending of AF of AE hit, with a relatively delayed trigger time.

Previous researchers determined the transition line by relying on the dominant frequency feature division and the test division [19,21,22], and used the polar initial movement method to verify the accuracy of the classification results [22]. On one hand, these empirical research methods are based on experience rather than physical mechanisms, which are limited by the discreteness and complexity of rocks. Besides, there exists a superposition of complex fracture types, and the AE frequency is also always affected by the fracture size. Although the transition line could accurately divide the typical tension and shear fractures, it is difficult to identify the mixed fracture types. On the other hand, the setting of waveform acquisition parameters during different experiment environments also causes the numerical difference of AE characteristic parameters. This should be considered in the search for universal standards to determine the transition line.

### 4.2. Analysis of Fracture Types Based on the Moment Tensor Method

**Figure 6:**
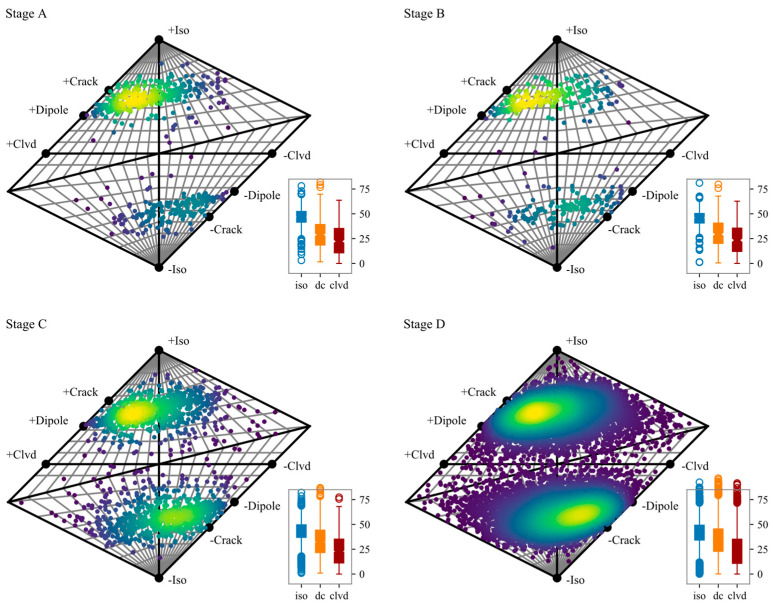
Hudson diagram of the final optimized moment tensor solutions. The horizontal axis describes the type of constant volume component in the source (DC vs. CLVD), while the vertical axis presents the proportion of the volumetric variations. The box plot at right bottom describes the statistics of the specific ratio of each component in the moment tensor decomposition.

The moment tensor of the events in each stage is calculated considering the uncertainties of P-wave polarity, azimuth, and the takeoff angles of the rays. The likelihood for the observations and measurement uncertainties is evaluated over a range of random moment tensors in a Bayesian framework. The final optimized moment tensor solutions are obtained and plotted in the Hudson diagram (Figure 6).

We draw the following conclusions from Figure 6. Firstly, there are obviously more crack event (+crack for tensile and -crack for compression) than shear (double couple, at the center of the Hudson diagram) events in these four stages. Secondly, there are more tensile events than compression events in the first three stages. Thirdly, the shear events began to increase in the stage D, but the corresponding proportion was still very small.

By comparing the statistics of the moment tensor decompositions of these four stages, it can be seen that the proportion of the ISO component decreases slightly as the loading increases (with mediums of 46.88, 46.06, 44.10, and 42.47, respectively). The shear composition gradually increases from 28.89% (medium) for stage A to 34.23% for stage D. There is no significant change for the CLVD component. We can draw a rough hypothesis here: the decreasing of ISO and the increment of DC can be used as a sign of rock crack development, and it may be a precursor of rock instability.

To verify the above hypothesis, we counted the number of events with shear components greater than a certain percentage at each stage (Table 2). When the DC component is greater than 40% in the moment tensor, the destructive effect of shear can no longer be ignored. It can be seen that in the first two loading stages, this type of events is about one-fifth, and it increases to more than one-third in the latter two stages. When the DC component is greater than 50%, the shear begins to play a leading role in the failure. It can be seen that, in the first two stages, this type of events counted about 6%, and it increases to 14.36% in the Stage C and to 17.09% in the Stage D. When the DC component is greater than 60%, the fracturing is dominated by shearing. This type of events varies from 2.81% in Stage A to 6.94% in Stage D. This shows that the increased shearing has the potential for predict instability.

Ma et al. [37] proposed a probabilistic model for classifying events, based on the event position in the Hudson diagram. According to the probabilistic model and the moment tensor inversion results of this study (Figure 6), the types of events are obtained (Table 3). The rock fracture evolution trends obtained by the 2 analysis methods are consistent, and the shear cracks or DC components are gradually increasing. However, the proportions of crack types in each stage are different in the results. This reflects two issues: firstly, the classification criteria for specific types are affected by experience; secondly, the 2 methods respectively classify cracks based on the initial movement direction of the p-wave and time-frequency characteristics, which results in different perspectives for extracting information from the AE waveform.

### 4.3. Uncertainty of the Moment Tensor Method

**Figure 7:**
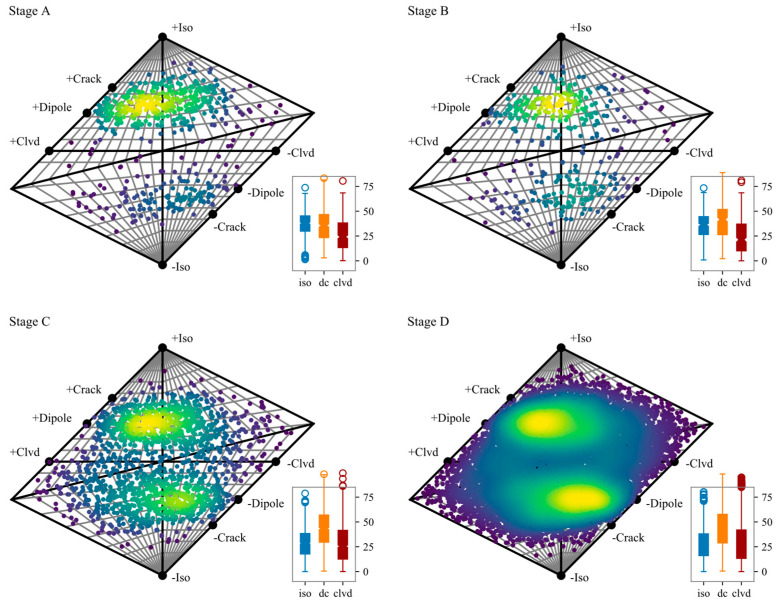
Hudson diagram of the final optimized moment tensor solutions consider only the first 6 triggered sensors. The box plot at right bottom describes the statistics of the specific ratio of each component in the moment tensor decomposition.

There is inevitable uncertainty using P-wave first motion to analyze the full moment tensor. The first aspect is the inaccuracy P-wave picking, and the spatial distribution of the sensors. The second aspect is that this method is essentially derived from earthquake fault analysis. The corresponding fault slip forms the tension and compression zones, and there is a difference in polarity. When it is generalized to the full moment tensor inversion, the uncertainty will increase, although the probability framework is adopted. In order to clarify the uncertainty caused by the inaccuracy of the polarity, we used the first six sensors to do another analysis, and the results are shown in Figure 7.

It can be seen through comparing Figure 6 and Figure 7 that when the constraint (the number of sensors used) is less, the shear component in the moment tensor inversion result will increase. The medium of shear component increased to 35.57, 41.65, 43.32, and 43.24 when only considering the first 6 triggered sensors. The proportion of events with DC component proportion larger than a certain value also increased (Table 4). It can also be seen from Figure 6 that when there are fewer constraints, the inversion results will be more scattered (Figure 7). When only the first 6 sensors are used, the same trends are observed: the effect of shearing on failure becomes greater as the loading increases.

### 4.4. Fracture Types of Granite in Post-Peak

**Figure 8:**
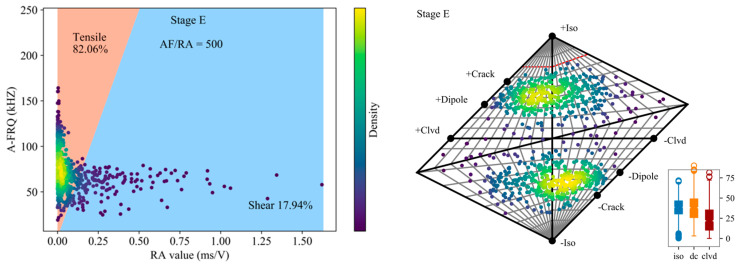
Classification of crack mode based on RA-AF value (using first-arrival AE hit) and Hudson diagram of the final optimized moment tensor solutions for the Stage E.

As indicated by the first four stages, shear-dominated events are gradually increasing with the instability intensifies. This phenomenon continues to Stage E (Figure 8). At this stage, the proportion of shear crack reach 21.50%, and the medium of the DC component is 37.93%, which continues to grow on the basis of the previous stage. Instead of absolutely classify the events into a certain source type, we should be concerned more about the proportions of individual components of the moment tensor and their evolutions over time.

**Figure 9:**
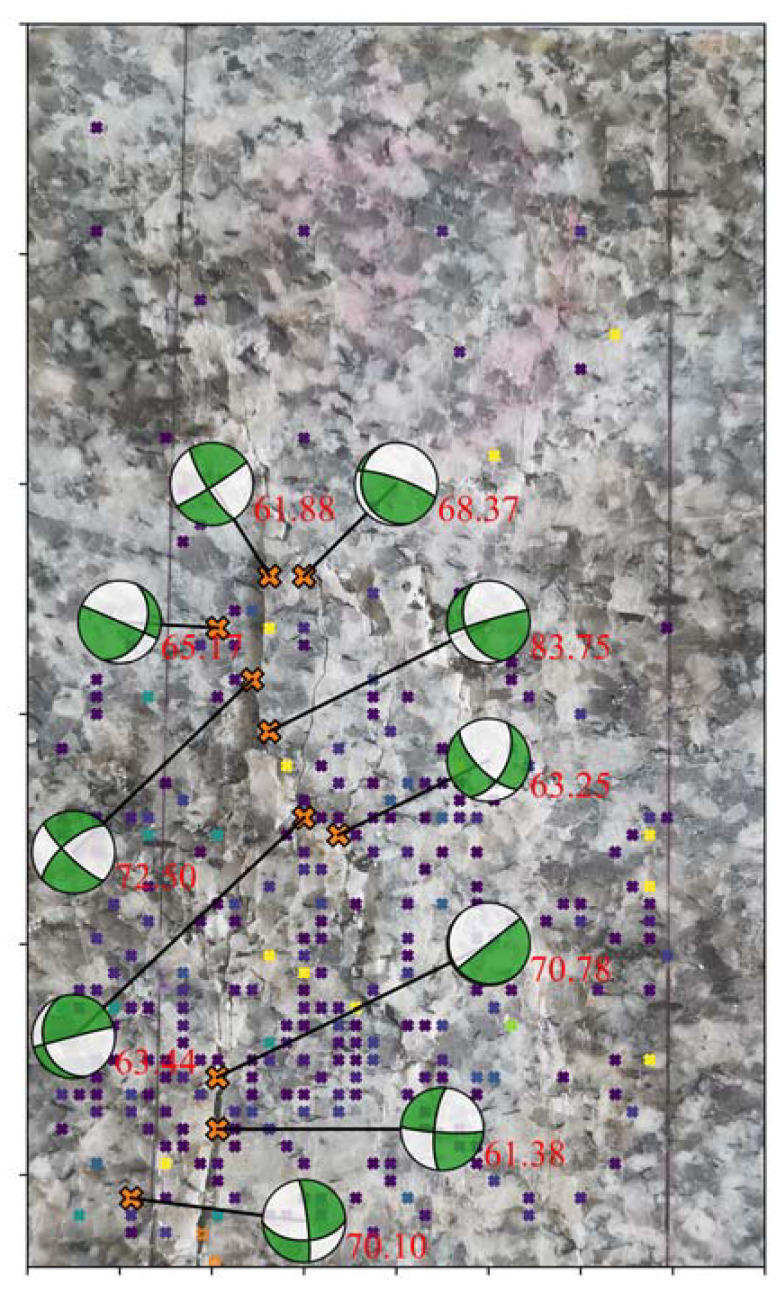
Events (in Stage E) with double couple component larger than 60% and their spatial position along the visible cracks.

In the last stage of loading (Stage E), the rock sample has shown visible macroscopic cracks. The positioning results of AE events are obtained by the localization method without using premeasured velocity based on the A* search algorithm [38]. We projected the events with DC components greater than 60% onto the rock sample (Figure 9) to analyze the effect of such events on crack development. In terms of spatial distribution, the shear-dominated events are all located near the visible cracks. We concluded that the failure is a process of event accumulation. In this process, shearing develops, from being unimportant to playing a leading role. Finally, under the action of shearing, the rock eventually fails.

## 5. Conclusions

(1) The proportion of shear cracks gradually increases during the whole loading process of uniaxial compression. The results demonstrate that the increasing of shear cracks is a significant sign of unstable crack propagation and coalescence. Water not only promotes the propagation of rock cracks, but also affects the fracture pattern of the rock.

(2) The evolution trends of rock cracks obtained by the 2 classification methods are consistent, but the difference in the analysis perspective of the AE signal causes the deviation of specific results.

(3) The RA-AF value distributions obtained using different AE hit data are different. The influence caused by the waveform acquisition configuration and the empirical thresholds of the different recognition methods is inevitable.

(4) Visible macroscopic cracks are mainly caused by shear fracturing. Instead of absolutely classifying the events into a certain source type, we need to pay more attention to the proportions of individual components of the moment tensor and their evolutions over time.

## Figures and Tables

**Table 1:** Comparison of fracture types between natural and saturated granites during evolution.

Sample	Method	Stage A [%]		Stage B [%]		Stage C [%]		Stage D [%]	
		Shear	Tensile	Shear	Tensile	Shear	Tensile	Shear	Tensile
Natural granite	AH	4.34	95.66	0.95	99.05	0.80	99.20	10.44	89.56
	FAH	2.28	97.72	0.27	99.73	0.64	99.36	13.11	86.89
Saturated granite	AH	5.86	94.14	3.10	96.90	0.84	99.16	6.25	93.75
	FAH	1.69	98.31	0.29	99.71	0.07	99.93	4.75	95.25

**Table 2:** The proportion of events with DC component proportion larger than a certain value (using all triggered sensors).

	Stage A [%]	Stage B [%]	Stage C [%]	Stage D [%]
DC Proportion > 60%	2.81	2.30	5.05	6.94
DC Proportion > 50%	6.19	6.62	14.36	17.09
DC Proportion > 40%	23.63	26.51	32.57	36.64

**Table 3:** The source types classified based on the event position in the Hudson diagram (using all triggered sensors).

	Stage A [%]	Stage B [%]	Stage C [%]	Stage D [%]
Explosive dominated	0.37	0	0.39	0.25
Tensile dominated	64.35	61.09	47.20	44.07
Shear dominated	3.75	6.05	10.50	14.55
Compression dominated	31.14	32.56	41.62	40.88
Implosive dominated	0.37	0.28	0.26	0.21

**Table 4:** The proportion of events with DC component proportion larger than a certain value (using only the first 6 triggered sensors).

	Stage A [%]	Stage B [%]	Stage C [%]	Stage D [%]
DC Proportion > 60%	8.44	14.98	20.07	21.58
DC Proportion > 50%	21.20	28.81	37.96	37.51
DC Proportion > 40%	38.46	51.58	56.58	56.18
